# Treatise on the Ear; Including Its Anatomy, Physiology, and Pathology

**Published:** 1840

**Authors:** 


### Treatise on the Ear; including its Anatomy, Physiology, and Pathology; (for which the Author obtained a Gold Medal in the University of Edinburgh). By *Joseph Williams*, M.D. Member of the Royal College of Surgeons.

The Essay before us obtained, as an Inaugural Dissertation, the recommendation of the Medical Faculty of Edinburgh to the Senatus Academicus, by whom a gold medal was awarded to the author. He modestly disclaims pretensions to a very complete account of so complicated a subject as the anatomy and pathology of the ear, and professes to have done little more than glance at the following topics:—The special Anatomy of the Human Ear—a brief description of Different Portions of the Ear in various Animals —the Theory and Laws of Sound—the Physiology of the Ear, and the Difficulties connected with that branch of the subject—the Pathology of the Ear, and some interesting Cases in illustration—Suggestions as to the mode of treating Diseases of the Ear—Instances of Morbid Alteration in Structure, and Congenital Malformations—their presence or absence in the congenitally Deaf and Dumb—and some observations respecting Medico-Legal facts connected with the Deaf and Dumb.

Aural Surgery has lately attracted, and is still attracting, so much attention from the profession, that we do not feel disposed to allow any opportunity of bringing it under the notice of our readers to escape us. We shall therefore run over the practical portion of the volume, and cull any facts or observations that may seem serviceable.

Dr. Williams first treats of the affections of the auricle, and notices in succession—idiopathic inflammation—erysipelas—acne—furuncle—herpetic eruptions—venereal blotches—scirrhus—wounds—malformation. We see nothing in his account of these to give us pause.

After noticing inflammation of the *meatus externus*, Dr. Williams alludes to the fact that the lining membrane of the meatus sometimes becomes thickened, and that to such a degree as to prevent the admission of vibration: this is generally observed in those who have had frequent attacks of inflammation of the meatus, with thin discharge and temporary deafness; the whole extent of the meatus is narrowed by thickening of the cuticle, with a thick white discharge resembling curds, with diminished or even suspended secretion of cerumen. Mr. Earle was the first to describe this peculiar thickening of the lining membrane of the meatus; and he proposes treating this disease by the application of the nitrate of silver. He mentions a case where a very strong solution of nitrate of silver was thrown in with a silver syringe, which completely blackened the epidermis of the meatus. In a few days, warm water was injected to loosen the exfoliations. They were detached in small portions at first, and subsequently in larger pieces, one of which, from its form, was evidently the reflected layer which covered the membrana tympani. After this, the injection of water caused a distressing sensation and loud noise. The other ear was served in a similar manner, and in a few days the hearing was very nearly restored. In a former number of this Journal we noticed this case of Mr. Earle's.

Dr. Williams makes a very good observation on the mode of EXAMINING the meatus.

"The auricle should be drawn upwards and backwards; and if the rays of the sun cannot be procured, an argand lamp must be used. Several useless inventions have come before the public at various times; a common argand lamp will answer every purpose, especially if a small concave reflector or mirror be attached. The speculum is of little use to *straighten* the canal, as it can exert no influence on the bony portion; and that part of the meatus capable of being straightened, may be effected by drawing the auricle upwards and outwards; besides, however delicately a pair of forceps may be made, they must take up a great part of the calibre of the canal. The multiplication of instruments forms no small part of the resources of the quack; and however unwilling he may be to explain their uses to the medical man, yet he takes good care that his patients shall not be ignorant that he possesses such weapons.

I may here mention that whenever a powerful stimulant is necessary to be applied to the meatus, let it be done by means of a camel-hair pencil; do not inject it, as you may run the risk of endangering the tympanum, and even in some cases the labyrinth." 117.

A *large meatus* is not favourable to acute hearing; and Itard is convinced, that far from increasing audition, it notably diminishes its extent and finesse. It has been proposed to remedy this defect by inserting a small leaden tube, exactly fitting the meatus; its external opening being covered by a piece of the swimming bladder of the fish, which should be stretched on while wet. In some cases, the power of hearing has been considerably augmented by the introduction of a gold tube.

*After Injuries of the Head*, a serous fluid, in large quantity, may escape from the meatus. It may or may not be preceded by blood. Dr. Williams believes, in all cases where this clear fluid escapes, there is fracture through the labyrinth; and hence arises a question as to whether this fluid is an increased secretion of the liquor of Cotunnius, whether serum of blood, or fluid from the membranes of the brain? It certainly is not the serum of blood, inasmuch as several ounces have drained away in the course of a few hours; and it has been tested, and was found not to be serum. In one instance, it was collected, and considerably exceeded a pint during the night.

Dr. Williams relates a case of death from puncture of the membrana tympani by a needle, and one of death from nitric acid being poured into the ear. In both cases, inflammatory action and consequent mischief took place in the tympanum, and affected the membranes of the brain.

After describing the symptoms, which cannot but be familiar, of inflammation of the tympanum, and observing, what is also known, that it may spread to the brain, he remarks that, under such circumstances it sometimes commences in the meatus auditivus externus, but it by far more frequently begins in the throat, passes through the Eustachian tube into the tympanum, thence to the internal ear, and then the brain becomes involved. This happens especially in those cases where the gastro-pulmonary mucous membrane is in an unhealthy condition; it occurs from small-pox, measles, and pharyngitis dependent upon disordered stomach, but more especially it arises from bad cases of scarlet fever.

It ought always to be borne in mind by the practitioner, that whenever there is severe pain in the ear, either with or without discharge, there *may* be danger of brain disease.

Dr. Williams adds :—

> "There is always danger when there is acute pain in the ear, *with pain and weight in the head,* especially if there be wandering of the eyes: and it is impossible to say when and how the inflammation may terminate, and therefore a cautious prognosis should always be given. Inflammation of the membranes of the brain is frequently an insidious disease, and a practitioner may easily be lulled into an idea of security, and imagine that he has merely a case of inflammation of the tympanum to treat, when meningitis actually exists. We should always suspect inflammation of the membranes when the pain, having previously been referred to the *ear*, suddenly *spreads* over the *head.*" 135.

When there are symptoms of this description, active antiphlogistic treatment is, of course, indicated. Dr. Williams very properly observes, that the system should be speedily brought under the influence of calomel; and, after the more acute stage, counter-irritants should be extensively and repeatedly applied. It often happens that persons attacked with internal otitis, which may have been very severe at its onset, but has been speedily arrested by general and local depletion, and by the application of blisters, &c. in such cases, especially if the general health has been much deranged for some time previously, relapses frequently recur, not sufficiently acute to demand the abstraction of blood, and yet the pain harasses the patient, *and not unfrequently extends to the forehead and back part of the head.* Here is the difficulty of distinguishing whether the membranes and brain are inflamed, or whether merely sympathetically affected; and the mere aurist, in such a case, must be a dangerous counsellor.

Dr. Williams goes on to remark, that abscesses sometimes form in the neighbourhood of the ear, caused by the irritation of diseased portions of the temporal bone, giving rise to symptoms resembling cerebral inflammation, for which they have occasionally been mistaken, leading to the abstraction of large quantities of blood, which has prostrated the powers of the patient, and sometimes induced death. Mr. Burne has mentioned such a case, where abscess was found behind the ear; the symptoms were attributed to phrenitis; active depletion was prescribed, extreme depression was the consequence, from which the patient did not rally, but gradually sank. Therefore it is necessary in all such cases carefully to examine and make pressure over the scalp, more particularly in the neighbourhood of the mastoid processes.

When injections are used, the patient often informs the medical attendant that, during the night, a clear discharge, like water, took place from the ear, leading the practitioner to suppose that caries exists; but this frequently arises from the injected fluid passing through a *small* ulcerated opening in membrana tympani, into the cavity of the tympanum, and which gradually drains off from the tympanum during the night, owing to the favourable position in which the head is placed for a lengthened period.

Our author very properly insists on the propriety of puncturing the membrana tympani, when the symptoms of abscess in the tympanum are decided and the suffering severe. For delay may lead to much mischief in the tympanum itself, or to extension of the inflammation to the mastoid cells, or even to the internal ear. The membrana tympani generally closes soon after the cessation of discharge.

Loss of the ossicula auditûs is a well known consequence of inflammation within the tympanum. The malleus and incus are much more easily displaced than the stapes, nor do they appear to be of the same importance. The whole of the ossicula have come away, and hearing has remained. A scrofulous child had suppuration of the internal ear, with loss of the whole of the ossicula, without injury to the sense of hearing; but generally, if the stapes be lost, deafness follows. When the ossicula are lost, and the immediate connecting medium between the external and internal ear is destroyed, patients hear better when the middle ear is filled with water.

When the tympanum has been destroyed, fungous excrescences frequently spring up, sometimes remaining concealed in the tympanum, at other times making their appearance in the meatus, and occasionally even protruding. The application of the sulphate of zinc will generally check their growth, and sometimes even effect their removal.

Speaking of *otorrhœa,* Dr. Williams says:—Chronic otorrhœa is frequently met with in scrofulous children; and in these cases the discharge usually comes from the meatus only, the glands being in a state of chronic inflammation. The discharge is almost always mucous, but occasionally, in severe cases, it is muco-purulent. It will generally run its course until the age of puberty, in spite of remedial means being adopted; sometimes it stops for a short time and then returns. But generally about the fourteenth or fifteenth year the discharge gradually becomes less and less, and is at last altogether suspended. It is better not to be over meddlesome with this affection. If suddenly checked in children, it frequently causes skin diseases, swelled glands in the neck, inflamed eyes, and sometimes brain affections. And the same caution applies to the sudden arrest of discharges from the ear in adults. Dr. Williams relates several instructive cases, calculated to impress this on our minds. The following observations cannot be too well attended to.

"Astringents, which ought *rarely* to be used, are highly dangerous when there is pain in the *head;* and if these injections be persevered in, the brain suffers and death generally follows. This renders the *quack specifics* so dangerous, as they are either composed of astringents, stimulants, or sedatives, each and all being decidedly injurious, when introduced into the tympanum, especially under such circumstances.

A girl had discharge from the ear; a *quack* (*charlatan*) injected into it an oily liquor, which caused considerable pain; inflammation succeeded, and she died delirious. Garlic, infused in oil of almonds and coloured by alkanet root, was a celebrated remedy for deafness.

Nothing is more common, when discharge is taking place from the ear, than to order these astringents; frequently without inquiring into *any* of the circumstances of the case. Whilst writing this thesis, I went to a public charity, with the *purpose* of seeing the treatment adopted in these cases. A woman presented herself, with purulent discharge from the external ear, which was of two weeks' standing; she complained of extreme pain in the *head;* she was ordered *instantly* an injection of the sulphate of zinc. I was unable to obtain any information respecting her subsequently.

In otorrhœa never use stimulating injections, for their presence has often produced violent inflammation of the tympanum, and even of the membranes of the brain, which has terminated in the death of the patient. In otorrhœa you can never be certain what disorganization has taken place in the internal ear, and therefore no one is warranted in injecting into it such dangerous agents."153.

The treatment that he recommends, judiciously consists of blisters, alteratives, and, in proper cases, tonics.

It occasionally happens, he observes, that the discharge ceases all at once, and this may arise from the meatus externus being blocked up; the matter speedily accumulates, and must be evacuated by breaking down with a probe the incrustations which have formed in the meatus. If dependent upon any internal obstruction, warm water should be gently injected, with the hope of washing it away.

The patient should always sleep on the affected side, to allow the matter by its gravity to drain off from the ear; and the ear should be syringed with tepid water night and morning. In these cases oil should never be introduced, as it speedily decomposes.

After some remarks on *otalgia*, Dr. Williams adverts to *caries*. Fortunately the mastoid process is more subject to it than the petrous. When present, the discharge is always sanious, highly offensive, and frequently discoloured; it stains a silver probe yellow, and very often minute portions of bone become detached, and pass out with the discharge. It is very important not to allow the matter to accumulate; the patient must therefore sleep on the affected side; must syringe the ear daily with warm water. The mastoid process should be frequently examined during otorrhœa, as instances have occurred where discharge has taken place, through the mastoid process, from the cells, and has burrowed under the cervical muscles, occasioning sloughing and death.

Perforation of the mastoid cells has been performed by two Swedish physicians, and there can be no doubt of the measure being in certain cases justifiable, indeed called for.

Inflammation of the brain and its membranes is apt to supervene on caries of the temporal bone. The dura mater in contact with it, inflames, separates from the bone, becomes discoloured, and pus is often deposited between it and the bone, or between it and the brain. The brain, sympathising with this membrane, becomes itself inflamed, and abscess takes place, which may or may not discharge by the ear. But sometimes it is not by continuous spreading of inflammation and its consequences that the brain becomes involved. Where otorrhœa has existed for some length of time, pus has been found in the brain, without any traces of communication between it and the ear being discovered, no caries of the petrous portion existing; so that there can be but little doubt, that extreme irritation and inflammation of the internal ear, is of itself sufficient to induce inflammation and suppuration of the brain. Dr. Alison has communicated to our author a case where abscess was found in the brain, consequent upon disease of the ear attended with discharge. The petrous portion was sound, consequently there was no communication between the matter of the ear and that of the brain. Dr. Alison has a drawing of the brain of this patient. Dr. Williams adds:—We often find irritation of the brain consequent upon teething; often inflammatory suppuration, depending upon the condition of the mucous membrane in fever; and nothing is more frequent than to find discharge from the ear, dependent upon gastric derangement: showing how great a sympathy exists between the brain, the ear, and the gastro-pulmonary membrane.

On the whole, the practitioner cannot be too much on his guard against cerebral mischief as a consequence of disease of the tympanum or internal ear. He should recollect, however, that neuralgia and paralysis of the face, with conjunctival inflammation, frequently occur independently of any disease of the brain, produced by a lesion of the portio dura. And he should also be aware that large collections of pus sometimes form under the pericranium in cases of severe otitis, leading one to suppose that mastoideal abscess has taken place. The free evacuation of matter will speedily relieve the patient, and very frequently, in the course of a few days, the discharge ceases, the mastoid process remaining perfectly sound.

*Affections of the membrana tympani* occupy our author next. Inflammation of it is usually the mere extension of inflammation of the meatus or of the tympanum. Laceration of the membrane has followed discharges of artillery—even a box on the ear—the incautious use of the probe—the removal of extraneous bodies from the meatus, &c. The membrane is much more frequently diseased than is generally imagined. Kramer mentions, that out of three hundred patients, thirty-five had chronic inflammation; and in twenty-eight of these it was partially destroyed, of which the practitioners who had previously attended the patients were not aware.

Leeches and antiphlogistic treatment are requisite to prevent thickening of the membrane, or ulceration.

"The membrana tympani rarely gives way in the centre, from the pressure of accumulated matter in the tympanum, but generally at one of its edges; in a few days, if the discharge cease, this membrane again closes. Valsalva performed several experiments on the membrana tympani of dogs; he ruptured this membrane and dilated it, and in a few days killed the animals, and found no traces whatever of the injury; and could not even discover the cicatrices. If the discharge continue a long time, and especially if the ossicula auditûs have come away, then the whole of this membrane becomes destroyed; and it is only in very rare cases that an artificial membrane is formed; but occasionally such membranes are produced, either in connexion with, or internal or external to, the membrana tympani. In speaking of false membranes covering the membrana tympani, Kramer seems to doubt whether they *ever* exist, and supposes that Fabricius Hildanus is the only person who has seen such a membrane. He imagined that Duverney was deceived, (who observed it in an adult,) by a partial abrasion of the membrana tympani." 178.

Mercury, given so as slightly to affect the system, is the remedy on which Dr. Williams principally relies. He adds:—an aperture in this membrane does not apparently diminish the power of hearing, and indeed it may be almost entirely lost, and yet partial hearing remain; formerly it was believed that deafness inevitably followed its destruction. When lacerated to any extent, near the attachment of the malleus, the patient is said to be unable to distinguish low or grave sounds.

It is often useful, but not always easy, to ascertain if there is an aperture in the membrana tympani. It may be generally seen by allowing the rays of the sun, or the reflected light of an argand lamp, to fall upon it: when in a healthy state it has a *tendinous* appearance, and when there is an aperture it appears as a *dark* spot. When inflamed it has a *dull brown* aspect, and numerous red vessels can be seen passing in every direction. If there is an opening, the lips and nostrils being closed, air passes out of the external meatus when forced from the mouth. The diagnosis may be further assisted by placing the finger gently upon the meatus when, if perfect, a low rumbling noise will be heard. Although simple, this will be found a very excellent mode of diagnosis. The rumbling noise is never heard when the membrana tympani is absorbed. If the aperture should be of any size, water and smoke will pass from the mouth, through the external meatus. Whenever the membrane is wanting, wool should be worn in the meatus.

When it becomes necessary to *puncture the membrana tympani*, a sharp-pointed silver probe is generally used, taking care to introduce it in that portion of the membrane anterior and inferior to its attachment with the malleus; or a sharp-pointed bistoury sliding through a canula might be preferred: this would cause considerably less pain if the membrane was much inflamed, as it always is when much pressed upon by an accumulation of purulent matter in the tympanum.

When a permanent opening is requisite, as in obliteration of the Eustachian tube, then a larger and a *circular* piece of membrane should be removed.

Dr. Williams gives the following directions for injecting the ear, when hardened cerumen accumulates in the meatus:—Care should be taken in choosing the syringe, which should not be small, as, although no force should be used, yet moderate power is frequently necessary. A syringe capable of containing two fluid ounces, will be found the most serviceable for washing out cerumen from the meatus. Some persons have recommended a size capable of containing only two or three drachms, but this is manifestly too small for such a purpose. It is important to have a short nossel, as the body of the syringe then forms a *guard* to prevent its entering too far into the meatus. Complicated instruments have been invented, but a common syringe with a short nossel will effect every purpose. It is convenient for those not wishing to increase the number of their instruments, to have a meatus shield fitted to an ordinary two or three ounce syringe. The nossel should not be so large as to completely fill up the meatus, otherwise the pressure might be too great, and laceration of the membrana tympani might take place, if the superabundant fluid could not pass out through the meatus. The piston should be pressed upon but feebly at first, and, if necessary, pressure may be gradually increased. If the injection cause *pain*, it should be immediately discontinued. It has been found that warm water dissolves the cerumen more readily than oil; but it has been recommended to drop a small quantity of oil into the meatus, the evening previous to injecting. It may be necessary to inject several times before the whole of the wax is washed out.

We would observe that hardened wax frequently escapes the notice of the surgeon and suspicion of the patient. We frequently see persons treated for affections of the head or for inflammatory conditions of the ear, in whom the sole disorder is a collection of cerumen in the meatus. We have always found it useful to have oil dropped into the ear night and morning, for two or three days before and after the syringing. A second syringing is generally necessary.

Dr. Williams enumerates the symptoms of *inflammation of the Eustachian tube* thus :—there is great pain in mastication and deglutition ; the patient is unable to force air into the tympanum, when the mouth and nostrils are closed : there is constant noise in the ear ; the tympanum is generally more or less involved ; and if the inflammation run high, the external meatus becomes painful upon pressure. The patient is unable to judge of the pitch or tone of his voice, and speaks in an unusual, and generally very loud key.

The antiphlogistic treatment, and more particularly emeto-cathartics, are required—gargles should be constantly used—and in some cases it will be advisable, as soon as the more acute inflammation has subsided, to inject warm water into the Eustachian tube, as it frequently happens that this tube becomes filled up with thick mucus, which, if allowed to remain, keeps up irritation, and, in the course of time, permanently obliterates this canal.

Dr. Williams makes the following judicious remarks on the operation of *injecting condensed air* through the Eustachian tube into the tympanum—an operation which has become but too notorious.

"It has lately been much advocated by some of our own countrymen ; and since this thesis was written, has been rendered but too public in consequence of its injudicious application. That the air acts on the tympanum as a very powerful stimulus is at once evident, from the numerous cases of inflammation which have been caused by its injection. It will require the sanction and experience of honest men, before we can admit of contradictory results to those obtained by M. Itard and others, who have not found it of use in a single case." 196.

Here we must close our notice of this fresh contribution to aural surgery. Dr. Williams has expended a good deal of labour and research upon the work, but we cannot compliment him on that lucidus ordo, and strict condensation which compilations should display. Practice will improve him, and we hope that a future edition will be characterised by the absence of what are very remediable defects.

